# Prediction of post-stroke cognitive impairment after acute ischemic stroke using machine learning

**DOI:** 10.1186/s13195-023-01289-4

**Published:** 2023-08-31

**Authors:** Minwoo Lee, Na-Young Yeo, Hyo-Jeong Ahn, Jae-Sung Lim, Yerim Kim, Sang-Hwa Lee, Mi Sun Oh, Byung-Chul Lee, Kyung-Ho Yu, Chulho Kim

**Affiliations:** 1 Department of Neurology, Hallym University Sacred Heart Hospital, Hallym University, Anyang, South Korea; 2 Department of Neurology, Chuncheon Sacred Heart Hospital, Hallym University, Chuncheon, South Korea; 3 Chuncheon Artificial Intelligence Center, Chuncheon Sacred Heart Hospital, Chuncheon, South Korea; 4 Department of Neurology, Asan Medical Center, University of Ulsan College of Medicine, Seoul, South Korea; 5 Department of Neurology, Kangdong Sacred Heart Hospital, Hallym University, Chuncheon, South Korea; 6 Institute of New Frontier Research Team, Hallym University College of Medicine, Chuncheon, South Korea

**Keywords:** Stroke, Dementia, Post-stroke cognitive impairment, Machine learning

## Abstract

**Background and objectives:**

Post-stroke cognitive impairment (PSCI) occurs in up to 50% of patients with acute ischemic stroke (AIS). Thus, the prediction of cognitive outcomes in AIS may be useful for treatment decisions. This PSCI cohort study aimed to determine the applicability of a machine learning approach for predicting PSCI after stroke.

**Methods:**

This retrospective study used a prospective PSCI cohort of patients with AIS. Demographic features, clinical characteristics, and brain imaging variables previously known to be associated with PSCI were included in the analysis. The primary outcome was PSCI at 3–6 months, defined as an adjusted *z*-score of less than − 2.0 standard deviation in at least one of the four cognitive domains (memory, executive/frontal, visuospatial, and language), using the Korean version of the Vascular Cognitive Impairment Harmonization Standards-Neuropsychological Protocol (VCIHS-NP). We developed four machine learning models (logistic regression, support vector machine, extreme gradient boost, and artificial neural network) and compared their accuracies for outcome variables.

**Results:**

A total of 951 patients (mean age 65.7 ± 11.9; male 61.5%) with AIS were included in this study. The area under the curve for the extreme gradient boost and the artificial neural network was the highest (0.7919 and 0.7365, respectively) among the four models for predicting PSCI according to the VCIHS-NP definition. The most important features for predicting PSCI include the presence of cortical infarcts, mesial temporal lobe atrophy, initial stroke severity, stroke history, and strategic lesion infarcts.

**Conclusion:**

Our findings indicate that machine-learning algorithms, particularly the extreme gradient boost and the artificial neural network models, can best predict cognitive outcomes after ischemic stroke.

**Supplementary Information:**

The online version contains supplementary material available at 10.1186/s13195-023-01289-4.

## Introduction

Post-stroke cognitive impairment (PSCI) refers to the development of cognitive deficits after index stroke in the absence of premorbid dementia and is one of the major determinants of functional dependence in post-stroke survivors [1]. The prevalence of PSCI ranges from 20 to 75%, according to ethnicity, country, post-stroke duration, and diagnostic criteria [2, 3]. PSCI not only causes cognitive impairment, but also increases the risk of other recurrent vascular events, including stroke [4, 5] and mortality [1]. Although the prevalence and burden of PSCI in stroke survivors are substantial, the prediction of PSCI development is still far from optimal.

Prediction of post-stroke cognition in patients with acute ischemic stroke (AIS) may be useful in deciding the course of cognitive assessment and treatment during the chronic care of patients with AIS. Previous studies have reported several prognostic scoring systems based on the clinical and/or radiological findings of patients with AIS. Two scoring systems, the CHANGE [6] and SIGNAL2 scale [7], have been shown to be modestly accurate in the prediction of PSCI, with areas under the receiver operating characteristic curve ranging from 0.740–0.829. As the pathophysiology and trajectory of cognitive decline after stroke are complex, with numerous determinants, traditional scoring systems with a limited number of variables may not optimally predict PSCI. Machine learning algorithms can easily incorporate numerous variables [8], including demographic, clinical, and imaging parameters, and may better predict PSCI.

Thus, we aimed to develop and determine the applicability of the machine learning (ML) models to predict PSCI after AIS. Furthermore, we analyzed and demonstrated the feature importance of input variables to determine the variables that are the most important PSCI Predictors.

## Methods

### Standard protocol approvals, registrations, and patient consent

This retrospective observational study was based on data from a prospective acute stroke registry. During hospitalization, written informed consent was obtained from all participants or their legal representatives for the use of clinical and imaging data in the prospective stroke registry [9]. Additional approval for this study, with a waiver for patient consent, was obtained from the Institutional Review Board of Hallym University Sacred Heart Hospital because of its retrospective nature and minimal risk to participants (IRB No. 2022–01-010–001).

### Study design and population

Consecutive patients with acute ischemic stroke admitted to a tertiary academic hospital within seven days of symptom onset were eligible to be enrolled in the study. All patients underwent standard evaluation and management according to the institutional stroke protocol, based on international and domestic guidelines. In addition to laboratory and imaging studies, a neuropsychological battery was conducted in patients with acute ischemic stroke 3 to 6 months after stroke onset who complained of cognitive decline or were at high risk for PSCI at the discretion of the attending physician [10].

The inclusion criteria for this study were as follows: (1) consecutive ischemic stroke patients from January 2011 to December 2020, (2) a relevant ischemic lesion observed on diffusion-weighted images, (3) admission within 7 days of symptom onset, and (4) available neuropsychological battery data 3 to 6 months after stroke onset. The participants were excluded if (1) they had a history of premorbid cognitive decline (i.e., those previously diagnosed with dementia and prescribed anti-cholinesterase inhibitors or memantine), (2) patients with a pre-stroke modified Rankin scale score of > 2, and (3) patients who were unable to participate in the neuropsychological tests due to hearing difficulty, poor cooperation, or neurological deficits including severe aphasia that would preclude the performance of neuropsychological tests.

### Clinical variables

We collected data on baseline and demographic factors, including age, sex, and education level at admission. Clinical factors included the initial National Institute of Health Stroke Scale (NIHSS) score and stroke subtype according to the Trial of ORG 10172 in Acute Stroke Treatment (TOAST) classification. Data on vascular risk factors, including arterial hypertension, dyslipidemia, diabetes mellitus, smoking status, previous history of stroke or transient ischemic attack, and potential sources of cardiac embolism, including atrial fibrillation, were collected. Laboratory results, including initial random glucose, white blood cell, total cholesterol, low-density lipoproteins, high-density lipoproteins, triglycerides, and creatinine, were also collected. All participants underwent brain magnetic resonance imaging using either a 1.5-T or 3-T whole-body magnetic resonance imaging system according to their year of admission. The laterality, multiplicity, and volume of the ischemic stroke lesions were collected. Furthermore, lesion locations were categorized as cortical, subcortical, or infratentorial. Strategic lesion locations were defined as the basal ganglia, thalamus, hippocampus, caudate nucleus, inferomedial temporal gyrus, and angular gyrus. Underlying small vessel diseases were evaluated based on the presence of lobar or deep chronic microbleeds and the degree of white matter hyperintensities according to the modified Fazekas scale [11]. Furthermore, the degree of mesial temporal lobe atrophy was determined using Schelten’s scale [12].

### Post-stroke cognitive impairment

The primary outcome was defined as the diagnosis of PSCI 3–6 months after stroke using the domain-specific definition. The PSCI was defined as having a standardized *z*-score of less than or equal to two standard deviations in at least one cognitive domain from the following: memory, language, visuospatial, and frontal/executive function. All participants were evaluated with a 60 min neuropsychological battery using the Korean version of the Vascular Cognitive Impairment Harmonization Standards-Neuropsychological Protocol (K-VCIHS-NP) at 3 to 6 months after stroke onset. The K-VCIHS-NP comprises four major cognitive domains, and the details of the included neuropsychological tests have been previously reported [3]. We also used the Korean version of the Mini-Mental State Examination (K-MMSE) to evaluate general cognitive function. All cognitive batteries in the K-VCIHS-NP were validated for use in the Korean population, and the scores of each test were transformed into *z*-scores after adjusting for age, sex, and years of education. Domain-specific *z*-scores were calculated using the average *z*-scores of each cognitive test comprising the domain-targeted tests. Pre-stroke cognitive assessments of the participants were performed with a structured questionnaire using the Korean version of the Informed Questionnaire on Cognitive Decline in the Elderly (IQCODE). IQCODE scores over 3.6 were set as a cutoff for premorbid cognitive decline. Secondary outcomes included the diagnosis of PSCI according to *z*-scores and raw K-MMSE scores. PSCI-MMSEz was diagnosed when the *z*-score of MMSE was less than − 2 SD and PSCI-MMSE was diagnosed when the raw MMSE score was less than 24.

### Machine learning model development

A total of 31 clinical and imaging variables were included in the ML model development for the prediction of PSCI (Supplemental Table 1). We used four ML algorithms: logistic regression [13], support vector machine (SVM) [14], extreme gradient boosting (XGB) [15], and artificial neural network (ANN)  [16]. Logistic regression is used for binary classification by substituting a linear function into a sigmoid function and expressing the result as 0 or 1. Support Vector Machine finds a hyperplane for binary classification, which is expressed in a high dimension according to the data input. Boosting is one of the ensemble techniques, and it is a model that improves errors by assigning weights to unpredicted data in the process of sequentially learning multiple weak learners. XGB is an ensemble model of weak learners, which is a decision tree that uses gradient descent to update the weights. Additionally, a technique to prevent overfitting was applied to the algorithm to improve the loss. An ANN is composed of several feedforward neural networks. In general, it is primarily used for the nonlinear classification of very complex problems, and its performance increases as the number of layers or variables increases. However, an excessive number of layer compositions and the use of multiple variables can cause overfitting. In this study, stratified k-fold, class weight, and random search techniques were used to prevent overfitting and model optimization. First, we divided the dataset into a training dataset and a test dataset in an 8:2 ratio, with 950 and 191 participants in each, respectively. Then, the training dataset was divided 10-fold, and cross-validation was performed by composing the same ratio of classes in the divided dataset. Cross-validation can prevent overfitting of a specific dataset and create a more generalized model. Among class weight, focal loss, and resampling techniques for solving the data imbalance problem, we applied the class weight technique. The ratio of the class of each outcome variable divided according to the MMSE score, MMSE *z*-score, and PSCI *z*-score was calculated to contribute equally to the loss calculation. While the grid search technique is generally used to search for hyper-parameters, we used random search [17] for better performance in finding the optimal hyper-parameters. The ML model was optimized using Optuna [18], a hyperparameter optimization framework based on random search.

Recently, as the performance of ML models has increased, the importance of XAI (eXplainable AI), which explains the results of the model, is increasing. Among them, we utilized SHAP (SHapley Additive exPlanations) [19] to express the feature attribution numerically. Specifically, feature attribution must satisfy local accuracy, missingness, and consistency. The SHAP values were the only additive feature importance measures that satisfied these three characteristics. In addition, the influence of the model was calculated by considering the dependence between variables. Therefore, it is possible to intuitively check the contribution of each variable in predicting PSCI. Thus, the feature importance and relationship of the PSCI-related variables were derived using SHAP values.

Among the study population, 80% were randomly selected for the training dataset and the remaining 20% were used as the test dataset. TensorFlow version 2.6.0, and scikit-learn version 1.0.2 were used for the training of the models. For hyperparameter tuning, Optuna 2.10.0 version was used. And Shap 0.40.0 version was used to calculate SHAP values.

### Statistical analysis

For the descriptive analysis, continuous variables are presented as mean ± standard deviation or medians with an interquartile range (IQR), as appropriate, and categorical variables with numbers and frequencies. Baseline, clinical, and imaging characteristics between the PSCI and no PSCI groups were compared using the *t*-test or Mann–Whitney *U* test for continuous variables and the chi-squared test or Fisher’s exact test for categorical variables, as appropriate. The area under the curve (AUC), accuracy, and F1 score were calculated to assess the performance of the developed ML models.

### Data availability statement

The data supporting the findings of this study are available from the corresponding author upon reasonable request.

## Results

### Baseline characteristics

**Fig. 1:**
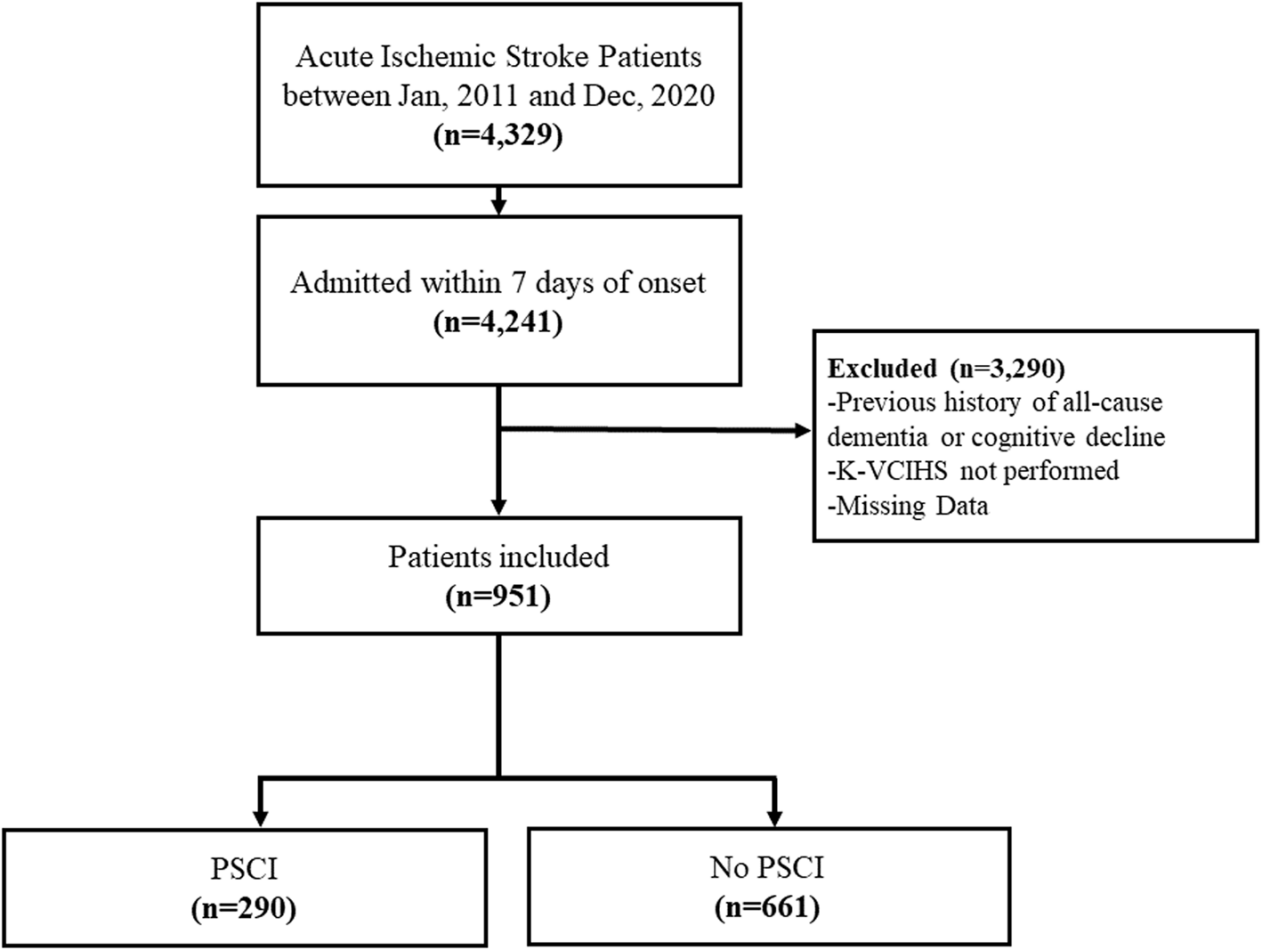
Study enrollment process. PSCI, post-stroke cognitive impairment. K-VCIHS, Korean version of Vascular Cognitive Impairment Harmonization Standard

Among the 4329 patients admitted with acute stroke during the study period, 951 patients were included in this study (Fig. 1). The mean age was 65.7 ± 11.9 years and the average interval between stroke onset to neuropsychological assessment was 4 months. The median NIHSS score was 2 (IQR 1–5) in our cohort.

Of the 951 patients included, 286 (30.1%) developed PSCI–3–6 months after stroke according to the K-VCIHS-NP results. The baseline characteristics of the PSCI and non-PSCI groups are shown in Table 1. The development of PSCI was significantly associated with older age, cardioembolic etiology, higher initial NIHSS score, larger stroke volume, and presence of cortical or strategic lesions. The PSCI group also had a more frequent history of hypertension, diabetes mellitus, coronary heart disease, atrial fibrillation, previous history of stroke or TIA, and higher levels of fasting blood glucose and MTLA scores.

**Table 1:** Demographic and clinical characteristics according to the status of post-stroke cognitive impairment

	Patients without PSCI (*n* = 661)	Patients with PSCI (*n* = 290)	*p*-value
**Demographic characteristics**			
Age, mean ± SD	64.9 ± 12.2	67.3 ± 11.1	**0.002**
Sex, male, *n* (%)	446 (62.5%)	198 (59.5%)	0.388
Education years, median [IQR]	9.0 [6.0; 12.0]	9.0 [6.0; 12.0]	0.651
Previous mRS, median [IQR]	0.0 [0.0; 0.0]	0.0 [0.0; 0.0]	**0.009**
BMI, mean ± SD	24.3 ± 3.1	23.9 ± 3.4	0.056
**Stroke characteristics**			
TOAST classification			**< 0.001**
LAA, *n* (%)	254 (35.6%)	125 (37.5%)	
SVO, *n* (%)	300 (42.0%)	93 (27.9%)	
CE, *n* (%)	82 (11.5%)	67 (20.1%)	
UD and OD, *n* (%)	78 (10.9%)	38(14.4%)	
Initial NIHSS, median [IQR]	2.0 [1.0; 4.0]	3.0 [1.0; 6.0]	**< 0.001**
Thrombolysis			0.057
IV tPA, *n* (%)	66 (9.2%)	38 (11.4%)	
IA thrombectomy, *n* (%)	9 (1.3%)	4 (1.2%)	
Combined IV + IA, *n* (%)	8 (1.1%)	11 (3.3%)	
**Lesion characteristics**			
Stroke volume (mm^3^), median [IQR]	4.2 [0.8; 20.5]	10.3 [1.3; 114.8]	**< 0.001**
Left-sided lesions, *n* (%)	402 (56.3%)	192 (57.7%)	0.73
Multiple lesions, *n* (%)	67 (9.4%)	34 (10.2%)	0.757
Cortical lesions, *n* (%)	221 (31.0%)	171 (51.4%)	**< 0.001**
Subcortical lesions, *n* (%)	362 (50.7%)	161 (48.3%)	0.625
Infratentorial lesions, *n* (%)	201 (28.2%)	65 (19.5%)	**0.004**
Strategic lesions, *n* (%)	229 (32.1%)	140 (42.0%)	**0.002**
**Vascular risk factors**			
Hypertension, *n* (%)	413 (57.8%)	215 (64.6%)	**0.046**
Diabetes mellitus, *n* (%)	191 (26.8%)	121 (36.3%)	**0.002**
Hyperlipidemia, *n* (%)	256 (35.9%)	108 (32.4%)	0.311
Previous stroke/TIA, *n* (%)	86 (12.0%)	68 (20.4%)	**0.001**
Coronary heart disease, *n* (%)	34 (4.8%)	27 (8.1%)	**0.044**
Atrial fibrillation, *n* (%)	74 (10.4%)	70 (21.0%)	**< 0.001**
Smoking, *n* (%)	277 (38.8%)	118 (35.4%)	0.329
**Laboratory findings**			
Total cholesterol (mg/dL), mean ± SD	181.3 ± 42.8	177.6 ± 46.9	0.217
Serum creatinine (mg/dL), mean ± SD	0.8 ± 0.5	0.9 ± 0.8	0.186
Hemoglobin (mg/dL), mean ± SD	14.0 ± 1.7	13.9 ± 1.7	0.24 3
Fasting blood sugar (mg/dL), mean ± SD	120.4 ± 42.6	129.0 ± 48.9	**0.006**
Systolic blood pressure (mmHg), mean ± SD	149.1 ± 25.8	146.8 ± 24.6	0.178
**Small vessel disease burden and atrophy**			
Microbleeds, *n* (%)	132 (18.5%)	67 (20.1%)	
Modified Fazekas score			0.086
mFS grade 0, *n* (%)	132 (18.5%)	51 (15.3%)	
mFS grade 1, *n* (%)	329 (46.1%)	144 (43.2%)	
mFS grade 2, *n* (%)	176 (24.6%)	85 (25.5%)	
mFS grade 3, *n* (%)	77 (10.8%)	53 (15.9%)	
Total MTLA, median [IQR]	2.0 [1.0; 3.0]	2.0 [2.0; 4.0]	**< 0.001**

*Abbreviations: PSCI* Post-stroke cognitive impairment, *BMI* Body mass index, *TOAST* Trial of ORG 10172 in Acute Stroke Treatment, *LAA* Large artery atherosclerosis, *SVO* Small vessel occlusion, *CE* Cardioembolism, *OD* Other determined, *UD* Undetermined, *NIHSS* National institute of health stroke scale, *IV* Intravenous, *tPA* tissue plasminogen activator, *IA* Intraarterial, mFS modified Fazekas scale, *MTLA* Medial temporal lobe atrophy

### Prediction models

**Fig. 2:**
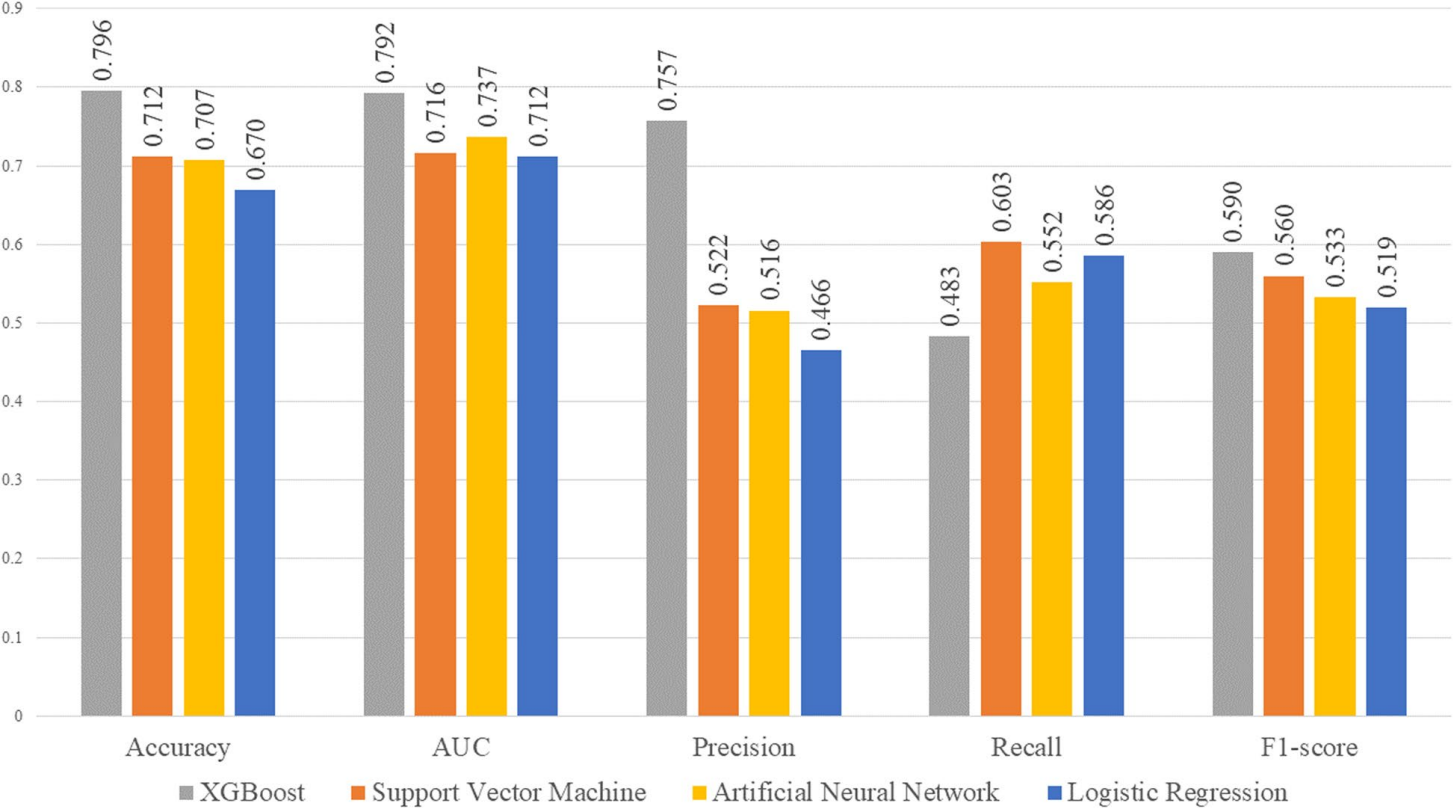
Comparison of machine learning model performance for the prediction of PSCI according to the VASCOG definition matrices of the best-performing model, XGB

**Fig. 3:**
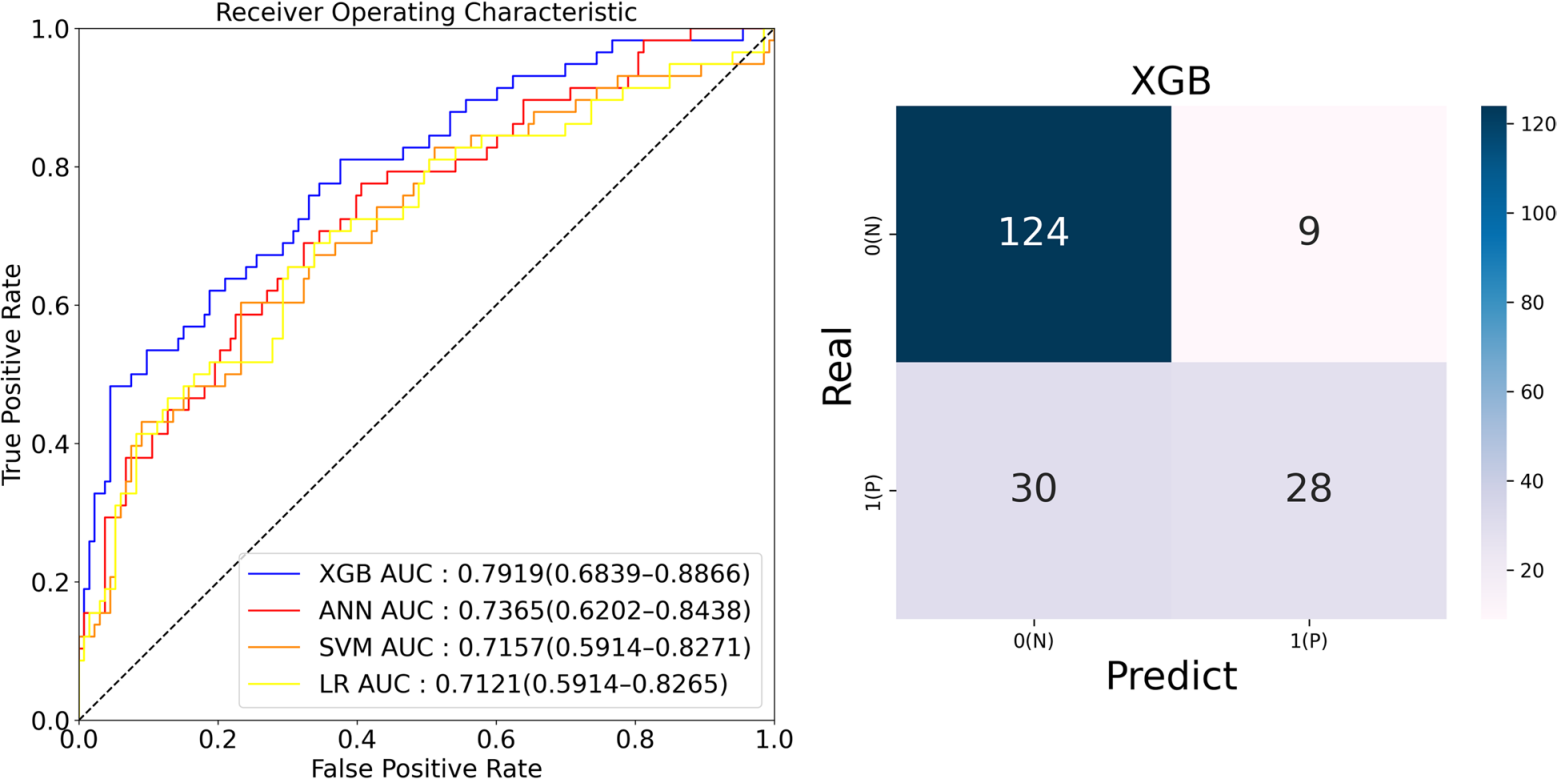
The receiver operating characteristic curves for the developed machine learning models and the confusion matrix of the best-performing model, XGB

Four models were developed for the prediction of PSCI, including logistic regression, SVM, XGB, and ANN models. The mean AUC for predicting PSCI was 0.7919 (0.6839–0.8866) for XGB, 0.7365 (0.6202–0.8438) for ANN, 0.7157 (0.5914–0.8271) for SVM, and 0.7121 (0.5914–0.8265) for logistic regression (Fig. 2 and Supplemental Table 2). The ROC curves for the best-performing folds and corresponding confusion matrices are shown in Fig. 3. The mean accuracy was the highest with XGB, followed by SVM, ANN, and logistic regression.

### Feature importance

**Fig. 4:**
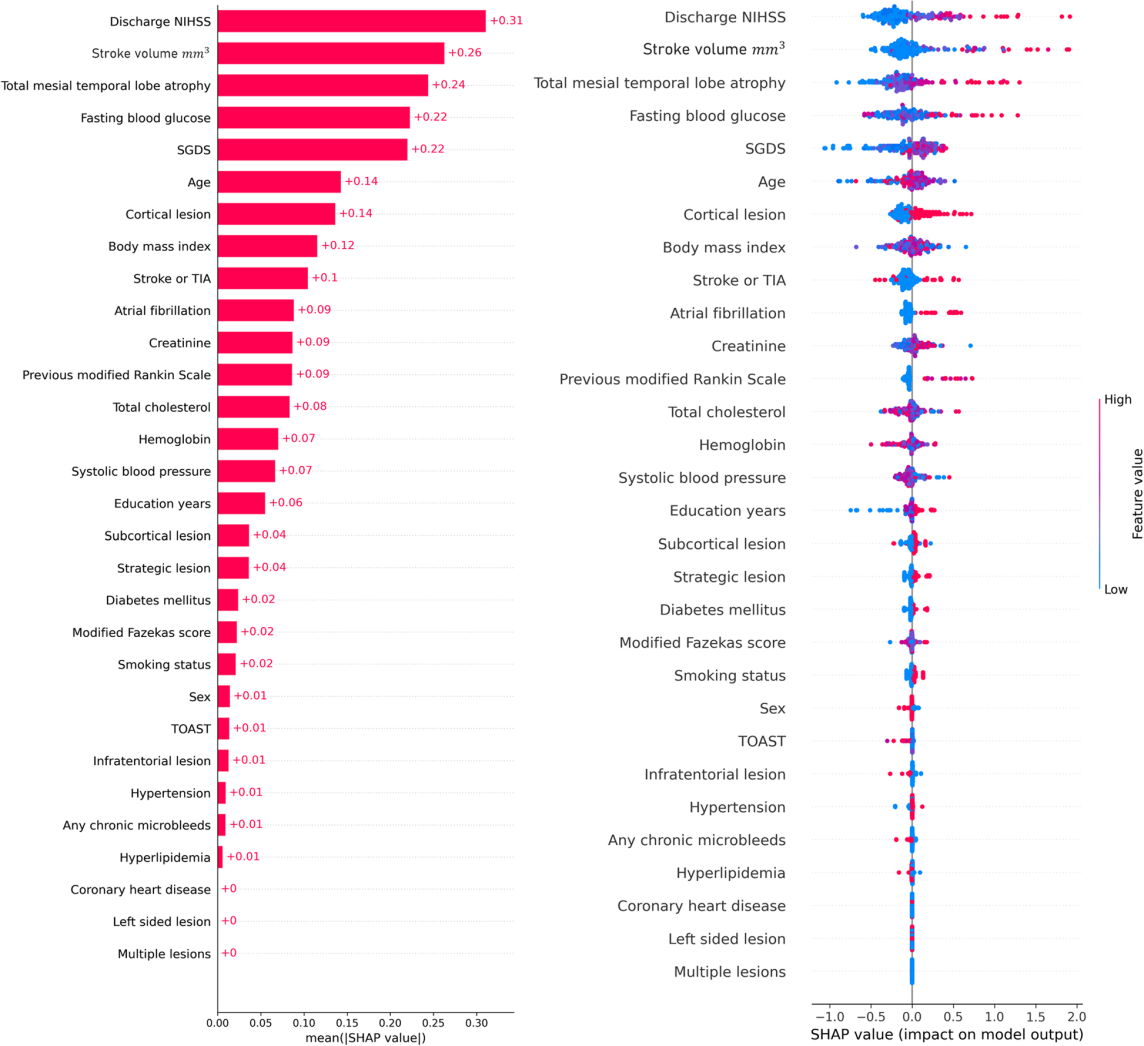
The SHapley Additive exPlanations values of the best prediction model, XGB

We determined the important variables used for the prediction of PSCI using the SHAP values of the best prediction model, the XGB (Fig. 4). The severity of the index stroke, assessed using the discharge NIHSS score and stroke volume, was the most important variable. Baseline medial temporal lobe atrophy was the second leading cause of PSCI development, followed by age, fasting blood sugar level, depression, age, and the presence of cortical lesions. History of previous stroke and atrial fibrillation was also utilized in the prediction model. However, the presence of left-sided lesions or multiple territory lesions was associated with zero SHAP values. Other common important features captured from three other ML models included cortical lesion, stroke severity, mesial temporal lobe atrophy, previous stroke, strategic lesion, and history of atrial fibrillation (Supplementary Fig. 1).

### Prediction models for the secondary outcomes

We used all four ML models for secondary outcomes with different diagnostic criteria for PSCI. The mean AUC for predicting PSCI-MMSEz was 0.7876 (0.6711–0.8892) for XGBoost, 0.7339 (0.6018–0.8525) for ANN, 0.7463 (0.6191–0.8566) for SVM, and 0.7608 (0.6434–0.8663) for logistic regression (Supplemental Table 3). The mean accuracy was the highest with XGB, followed by SVM, ANN, and logistic regression. The ROC curves for the best-performing folds are shown in Supplemental Fig. 2A. The overall AUC for the prediction of the PSCI-MMSE was the highest among the outcome variables. The mean AUC for predicting PSCI-MMSE was 0.8751 (0.7838–0.9472) on SVM, 0.8741 (0.8165–0.9241) on ANN, 0.8713 (0.7831–0.9414) on LR and 0.8616 (0.7683–0.9389) on XGBoost. The mean accuracy was the highest with ANN (0.8639), followed by XGBoost, SVM, and LR. The ROC curves for the best-performing folds are shown in Supplemental Fig. 2B.

## Discussion

We developed ML models to predict PSCI in patients with acute ischemic stroke. We demonstrated that the ML approach can accurately predict short-term cognitive outcomes after an acute stroke. Among the four ML models, XGB had the highest accuracy and largest area under the curve. Furthermore, the most important features associated with the prediction of PSCI include stroke severity, stroke volume, mesial temporal lobe atrophy, fasting blood glucose, age, and cortical lesions.

As the pathophysiology and contributing factors for the development of PSCI are diverse and complex [20], the prediction of PSCI is less accurate than the prediction of functional outcomes after stroke in clinical practice. Although multiple traditional prediction models [21] and ML models [22] for the prediction of functional outcomes after ischemic stroke have been reported with high accuracy, there are few prediction models for PSCI in the literature. Furthermore, this study is the first to incorporate ML techniques with both demographic and image variables to predict PSCI. Among the prediction models, Chander et al. reported the CHANGE (Chronic lacunes, Hyperintensities, Age, Non-lacunar cortical infarcts, Global atrophy, and Education) score using logistic regression models with PSCI at 3–6 months as the outcome. They used the cut-off raw score of either the Mini-Mental Status Examination (MMSE) ≤ 25 or Montreal Cognitive Assessment (MOCA) ≤ 22. The overall accuracy and area under the ROC curve for the model development cohort were 73.7% and 0.820, respectively. The SIGNAL_2_ model also used the definition of PSCI using the same cutoff values of MMSE and MoCA as the CHANGE model. The SIGNAL_2_ model also had an AUC of 0.829 for the prediction of PSCI using both clinical and neuroimaging variables. Meanwhile, the machine learning model we developed had an accuracy of 79.6% and an AUC of 0.792, which are relatively lower than those of traditional risk prediction models, whereas our models utilized more than 30 input variables with the ML approach. These discrepancies are mainly due to the fact that MMSE and MoCA scores are highly dependent on age and education level. Both the CHANGE and SIGNAL2 models used age and education as input variables with high weights; thus, the prediction of raw score cutoff of MMSE and MoCA may be higher regardless of patients’ clinical characteristics. In this regard, the AUC and accuracy were as high as 0.8751 and 81.7%, respectively, in the secondary outcome of our study using the raw MMSE score, which is higher than that of the traditional risk prediction models. However, as the diagnosis of PSCI is mostly based on the standardized *z*-scores of each neurocognitive domain in recent diagnostic criteria, including the VASCOG [23] and VICCCS criteria [24], the overall accuracy achieved with ML techniques using validated diagnostic criteria is more suitable than previous studies.

Recently, eXplainable Artificial Intelligence (XAI) [25] has been developed, in which investigators can understand the important variables that were utilized in the predictions made by AI. Among them, we performed Shapley Additive Explanations, which produce Shapley values for each input variable to measure the contribution to the prediction. Among 30 clinical and neuroimaging variables, the most important features for the prediction of PSCI in the best-performing models were stroke severity, stroke volume, mesial temporal lobe atrophy, age, fasting blood sugar, cortical lesions, body mass index, and history of previous stroke. The important features of other ML models that are not in the higher order in the XGB model include strategic infarction, history of hypertension, and depression. This order of feature importance is in accordance with previous risk factor studies on PSCI [10, 26, 27]. Previous studies on neuroimaging markers have revealed that the adjusted R^2^ for the prediction of PSCI was the highest for stroke volume, followed by total brain tissue volume, total medial temporal lobe atrophy, and the presence of strategic strokes. Further significant predictors with less meaningful *R*^2^ were a history of stroke, left hemispheric lesion, microbleeds, and white matter hyperintensity burden [28]. Old age, low educational level, history of hypertension, fasting blood sugar, and body mass index have also been reported as potential risk factors for stroke in previous studies [29–31].

Most of the important predictors for the models were unmodifiable factors such as age, previous stroke history, and stroke lesion characteristics. While vascular risk factors, including hypertension, dyslipidemia, diabetes, and atrial fibrillation also showed a strong association with PSCI development, there is limited evidence that controlling these modifiable risk factors would lower the incidence of PSCI [32]. Thus, it remains unclear whether the prediction of PSCI in the acute stroke stage may help prevent the development of PSCI. However, it may be helpful to perform a careful and thorough cognitive assessment at a reasonable time point after stroke in patients whose PSCI is predicted by the ML approach to effectively diagnose and improve cognitive status with potential therapeutic options at an earlier stage.

This study had several limitations. First, this was based on a single-center cohort and thus requires external validation. Although we only included clinical variables and imaging variables that are typically obtained or evaluated in most stroke centers, stroke registries with a routine cognitive assessment with a full neuropsychological battery are scarce. Second, the attrition rate was high in this cohort and mostly included patients with mild ischemic stroke who could complete neuropsychological batteries, thereby precluding the generalizability of our model. Thirdly, we utilized the MMSE as one of our study’s outcome variables in place of the MoCA. While the MoCA is recognized for its greater sensitivity and specificity in detecting cognitive decline in patients with PSCI [33], not all participants in our study undertook this test. To minimize selection bias, we chose to implement the MMSE. This decision also facilitated comparison with previous studies, which predominantly utilized MMSE as their outcome variables. Furthermore, the ML models are subject to improvement with additional features, including raw MRI images including DWI or DTI, which may represent network connectivity and other unknown imaging features associated with the development of PSCI.

## Conclusion

We demonstrated that ML models, particularly the XGB model, could accurately predict short-term cognitive outcomes after acute ischemic stroke. Among these variables, the most important features associated with the prediction of PSCI included stroke severity, stroke volume, mesial temporal lobe atrophy, fasting blood glucose, age, and cortical lesions. However, it remains to be determined whether accurate prediction of PSCI development can indeed contribute to mitigating cognitive decline in these patients.

### Supplementary Information

**Additional file 1: Supplemental Fig. 1.** The SHapley Additive exPlanations values of the machine learning models including ANN, SVM, and logistic regression for the prediction of PSCI using VASCOG criteria. **Supplemental Fig. 2.** Receiver Operating Characteristic curves for the developed machine learning models for the secondary outcomes (A) PSCI-MMSEz and (B) PSCI-MMSE.** Supplemental Table 1.** Input variables for machine learning model development. **Supplemental Table 2.** Comparison of machine learning model performance for the prediction of PSCI according to the VASCOG definition. **Supplemental Table 3.** Comparison of machine learning model performance for the prediction of secondary outcomes.

## Data Availability

The data that support the findings of this study are available on request from the corresponding author, CK.
