# Supplementary material for: Prediction of post-stroke cognitive impairment after acute ischemic stroke using machine learning
Source: Alzheimers Res Ther. 2023 Aug 31;15:147. doi: 10.1186/s13195-023-01289-4 (PMC10468853; doi:10.1186/s13195-023-01289-4)
Supplement: Supplementary file 1 — Additional file 1: Supplemental Fig. 1. The SHapley Additive exPlanations values of the machine learning models including ANN, SVM, and logistic regression for the prediction of PSCI using VASCOG criteria. Supplemental Fig. 2. Receiver Operating Characteristic curves for the developed machine learning models for the secondary outcomes (A) PSCI-MMSEz and (B) PSCI-MMSE. Supplemental Table 1. Input variables for machine learning model development. Supplemental Table 2. Comparison of machine learning model performance for the prediction of PSCI according to the VASCOG definition. Supplemental Table 3. Comparison of machine learning model performance for the prediction of secondary outcomes. [file 13195_2023_1289_MOESM1_ESM.zip › Supplemental Materials_revised.docx]

**Supplemental Materials**

**Supplemental Figure 1.** The SHapley Additive exPlanations values of the machine learning models including ANN, SVM, and logistic regression for the prediction of PSCI using VASCOG criteria.

**Supplemental Figure 2.** Receiver Operating Characteristic curves for the developed machine learning models for the secondary outcomes (A) PSCI-MMSEz and (B) PSCI-MMSE.

| **Supplemental Table 1. Input variables for machine learning model development** | |
| --- | --- |
| **Demographics** | Age |
|  | Sex |
|  | Body mass index |
|  | Education years |
|  | Previous modified Rankin Scale |
| **Vascular Risk factors** | History of hypertension |
|  | History of diabetes mellitus |
|  | History of hyperlipidemia |
|  | History of coronary heart disease |
|  | History of stroke or TIA |
|  | History of atrial fibrillation |
|  | Smoking status |
| **Stroke Clinical Characteristics** | Discharge NIHSS |
|  | TOAST classification |
| **Neuroimage characteristics** | Multiple lesions |
|  | Left sided lesions |
|  | Stroke volume (mm^3^) |
|  | Presence of cortical lesion |
|  | Presence of subcortical lesion |
|  | Presence of infratentorial lesion |
|  | Presence of strategic lesion |
|  | Modified Fazekas score |
|  | Any chronic microbleeds |
|  | Total mesial temporal lobe atrophy |
| **Laboratory results** | Fasting blood glucose |
|  | Creatinine |
|  | Total cholesterol |
|  | Hemoglobin |
|  | Systolic blood pressure |
| **Others** | SGDS |
| *Abbreviations: NIHSS; National Institute of Health Stroke scale, TOAST; Trial of ORG 10172 in Acute Stroke Treatment, SGDS; Short-form geriatric depression scale. | |

| **Supplemental Table 2. Comparison of machine learning model performance for the prediction of PSCI according to the VASCOG definition** | | | | | |
| --- | --- | --- | --- | --- | --- |
|  | **Accuracy** | **AUC** | **Precision** | **Recall** | **F1-score** |
| **XGB** | 0.7958 (0.7263-0.8632) | 0.7919 (0.6839-0.8866) | 0.7568 (0.5789-0.9375) | 0.4828 (0.3103-0.6552) | 0.5895 (0.4091-0.7451) |
| **SVM** | 0.7120 (0.6211-0.8000) | 0.7157 (0.5914-0.8271) | 0.5224 (0.4000-0.6667) | 0.6034 (0.4138-0.7586) | 0.5600 (0.4194-0.6885) |
| **ANN** | 0.7068 (0.6105-0.8000) | 0.7365 (0.6202-0.8438) | 0.5161 (0.3793-0.6667) | 0.5517 (0.3793-0.7241) | 0.5333 (0.3860-0.6769) |
| **LR** | 0.6702 (0.5789-0.7684) | 0.7121 (0.5914-0.8265) | 0.4658 (0.3514-0.6000) | 0.5862 (0.4138-0.7586) | 0.5191 (0.3824-0.6471) |

| **Supplemental Table 3. Comparison of machine learning model performance for the prediction of secondary outcomes** | | | | | |
| --- | --- | --- | --- | --- | --- |
|  | **Accuracy** | **AUC** | **Precision** | **Recall** | **F1-score** |
| **PSCI-MMSEz** | | | | | |
| **XGB** | 0.8115 (0.7579 – 0.8737) | 0.7876 (0.6711 – 0.8892) | 0.5789 (0.2727 – 0.8889) | 0.2821 (0.1053 – 0.4737) | 0.3793 (0.1538 – 0.5926) |
| **SVM** | 0.7068 (0.6105 – 0.8000) | 0.7463 (0.6191 – 0.8566) | 0.3836 (0.2759 – 0.5000) | 0.7179 (0.5263 – 0.8947) | 0.5000 (0.3636 – 0.6222) |
| **ANN** | 0.7644 (0.6947 – 0.8421) | 0.7339 (0.6018 – 0.8525) | 0.4062 (0.2000 – 0.6364) | 0.3333 (0.1579 – 0.5789) | 0.3662 (0.1600 – 0.5641) |
| **LR** | 0.6702 (0.5789 – 0.7579) | 0.7608 (0.6434 – 0.8663) | 0.3500 (0.2500 – 0.4474) | 0.7179 (0.5263 – 0.8947) | 0.4706 (0.3396 – 0.5862) |
| **PSCI-MMSE** | | | | | |
| **XGB** | 0.8272 (0.7789 – 0.8842) | 0.8616 (0.7683 – 0.9389) | 0.7500 (0.5000 – 1.0000) | 0.3488 (0.1429 – 0.5714) | 0.4762 (0.2308 – 0.6667) |
| **SVM** | 0.8168 (0.7368 – 0.8947) | 0.8751 (0.7838 – 0.9472) | 0.5690 (0.4333 – 0.7200) | 0.7674 (0.5714 – 0.9524) | 0.6535 (0.5098 – 0.7826) |
| **ANN** | 0.8639 (0.8168 - 0.9058) | 0.8741 (0.8165 - 0.9241) | 0.7576(0.6279 – 0.8929) | 0.5814(0.4419 – 0.7209) | 0.6579 (0.5278 – 0.7727) |
| **LR** | 0.8063 (0.7263 – 0.8842) | 0.8713 (0.7831 – 0.9414) | 0.5536 (0.4138 – 0.7037) | 0.7209 (0.5238 – 0.9048) | 0.6263 (0.4783 – 0.7556) |
