# Supplementary figures and images for: Prediction of post-stroke cognitive impairment after acute ischemic stroke using machine learning
Source: Alzheimers Res Ther. 2023 Aug 31;15:147. doi: 10.1186/s13195-023-01289-4 (PMC10468853; doi:10.1186/s13195-023-01289-4)

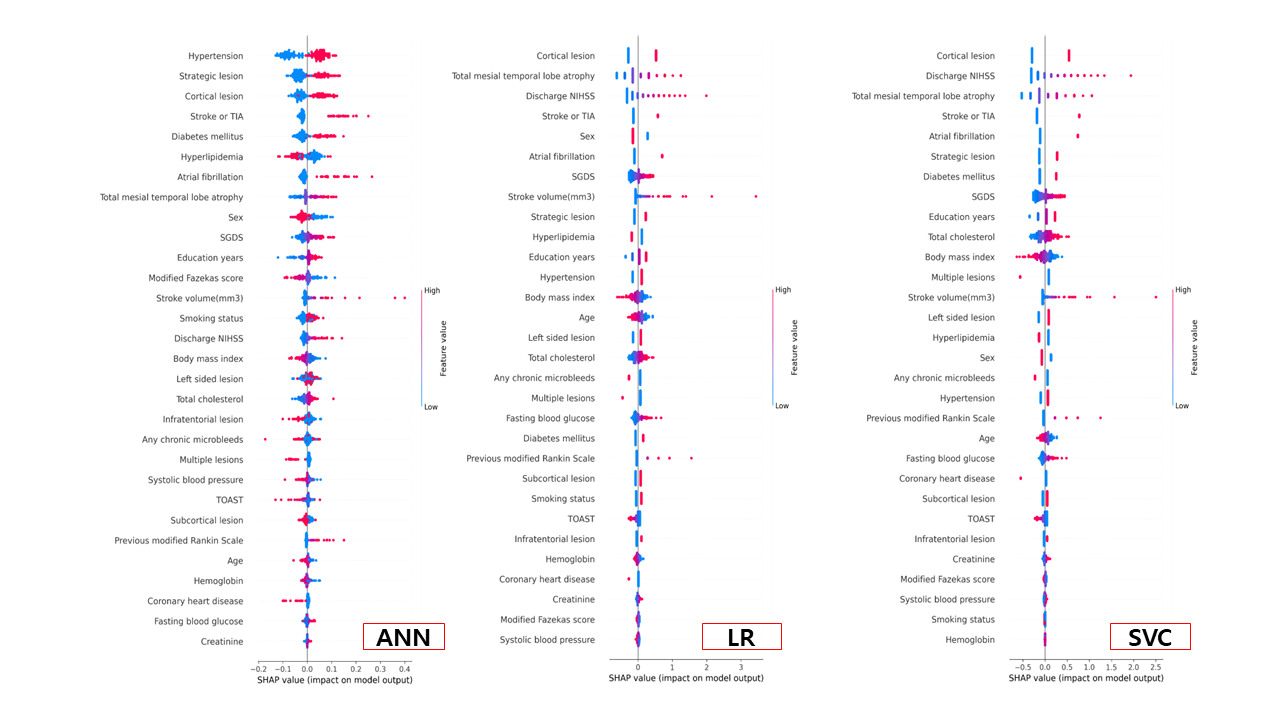

Supplement: Supplementary file 1 — Additional file 1: Supplemental Fig. 1. The SHapley Additive exPlanations values of the machine learning models including ANN, SVM, and logistic regression for the prediction of PSCI using VASCOG criteria. Supplemental Fig. 2. Receiver Operating Characteristic curves for the developed machine learning models for the secondary outcomes (A) PSCI-MMSEz and (B) PSCI-MMSE. Supplemental Table 1. Input variables for machine learning model development. Supplemental Table 2. Comparison of machine learning model performance for the prediction of PSCI according to the VASCOG definition. Supplemental Table 3. Comparison of machine learning model performance for the prediction of secondary outcomes. [file 13195_2023_1289_MOESM1_ESM.zip › Supplemental Figure 1.png]

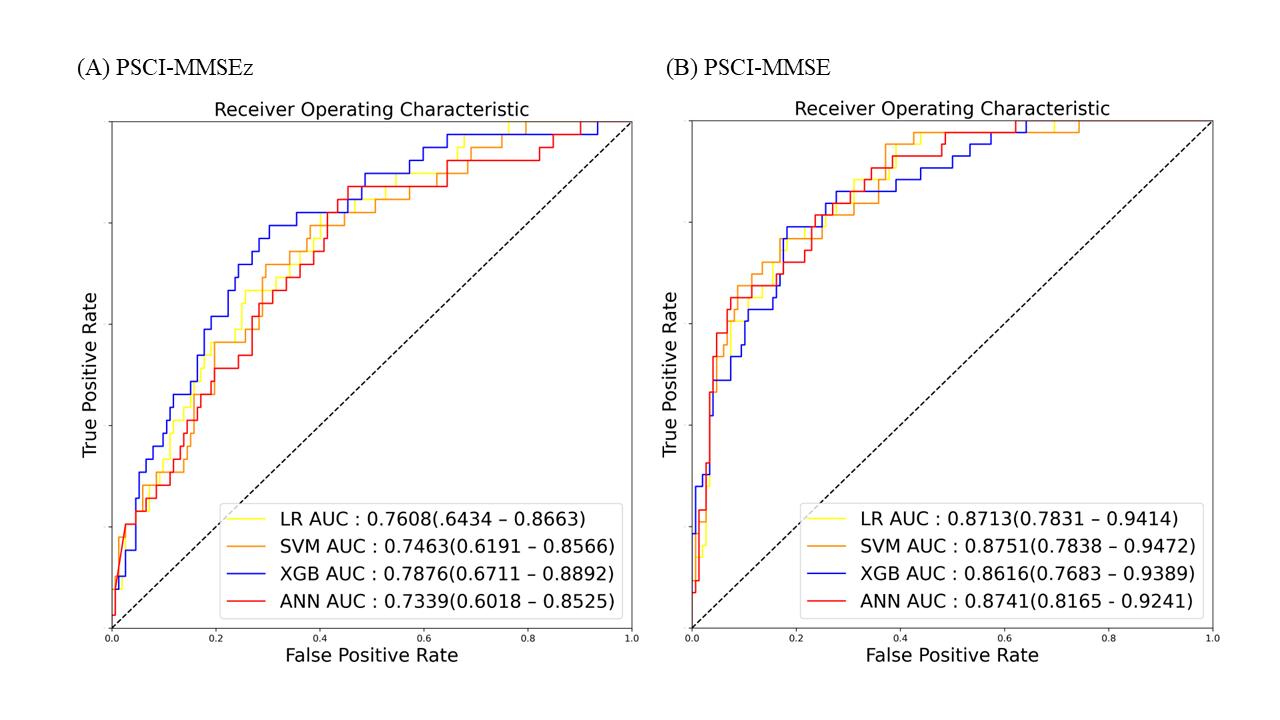

Supplement: Supplementary file 1 — Additional file 1: Supplemental Fig. 1. The SHapley Additive exPlanations values of the machine learning models including ANN, SVM, and logistic regression for the prediction of PSCI using VASCOG criteria. Supplemental Fig. 2. Receiver Operating Characteristic curves for the developed machine learning models for the secondary outcomes (A) PSCI-MMSEz and (B) PSCI-MMSE. Supplemental Table 1. Input variables for machine learning model development. Supplemental Table 2. Comparison of machine learning model performance for the prediction of PSCI according to the VASCOG definition. Supplemental Table 3. Comparison of machine learning model performance for the prediction of secondary outcomes. [file 13195_2023_1289_MOESM1_ESM.zip › Supplemental Figure 2.png]
